# Anti-Colorectal Cancer Effects of Probiotic-Derived p8 Protein

**DOI:** 10.3390/genes10080624

**Published:** 2019-08-19

**Authors:** Byung Chull An, Sunwoong Hong, Ho Jin Park, Bong-Kyu Kim, Jun Young Ahn, Yongku Ryu, Jae Hyung An, Myung Jun Chung

**Affiliations:** R&D Center, Cell Biotech, Co., Ltd, 50, Aegibong-ro 409 beon-gil, Gaegok-ri, Wolgot-myeon, Gimpo-si, Gyeonggi-do 10003, Korea

**Keywords:** *Lactobacillus rhamnosus* KCTC 12202BP, probiotics, therapeutic protein, anti-cancer activity, p8, drug delivery system, gene therapy

## Abstract

Recently, we reported a novel therapeutic probiotic-derived protein, p8, which has anti-colorectal cancer (anti-CRC) properties. In vitro experiments using a CRC cell line (DLD-1), anti-proliferation activity (about 20%) did not improve after increasing the dose of recombinant-p8 (r-p8) to >10 μM. Here, we show that this was due to the low penetrative efficiency of r-p8 exogenous treatment. Furthermore, we found that r-p8 entered the cytosol through endocytosis, which might be a reason for the low penetration efficiency. Therefore, to improve the therapeutic efficacy of p8, we tried to improve delivery to CRC cells. This resulted in endogenous expression of p8 and increased the anti-proliferative effects by up to 2-fold compared with the exogenous treatment (40 μM). Anti-migration activity also increased markedly. Furthermore, we found that the anti-proliferation activity of p8 was mediated by inhibition of the p53-p21-Cyclin B1/Cdk1 signal pathway, resulting in growth arrest at the G_2_ phase of the cell cycle. Taken together, these results suggest that p8 is toxic to cancer cells, shows stable expression within cells, and shows strong cancer suppressive activity by inducing cell cycle arrest. Therefore, p8 is a strong candidate for gene therapy if it can be loaded onto cancer-specific viruses.

## 1. Introduction

In 2018, an estimated 145,600 adults in the United States were diagnosed with colorectal cancer (CRC) and there were an estimated 51,020 deaths. The 5-year survival rate in the United States is around 65% [1]. Colorectal cancer is a cancer of the intestine that can invade or spread to other parts of the body [2]. Treatments include a combination of surgery, radiation therapy, chemotherapy, and targeted therapy [3]. Chemotherapy for CRC involves natural, synthetic, or biological substances that suppress or prevent progression. However, many chemotherapy agents are toxic to normal cells [4]. 

To identify new biotherapeutic drugs with fewer/less severe side effects, many studies have screened probiotics [5,6,7]. Because human intestinal microbes and probiotics are generally regarded as safe, isolated proteins may have anti-CRC effects but may show reduced systemic toxicity [8,9,10]. Indeed, a probiotic-derived protein that suppresses CRC would likely have few adverse effects [11,12]. Generally, food-grade bacteria are (by definition) safe to ingest [8]. Historically, such microbes have not been associated with the development of sinister pathologies; indeed, their positive impact on health is well documented in the context of human and animal food production [12]. Thus, we can conclude (albeit with a degree of caution) that probiotic-derived proteins are relatively safe. 

An et al. [13] screened laboratory strains of probiotics (all originating from the human intestine) to identify novel therapeutic proteins against CRC. The screening process identified an 8 kDa protein (p8) isolated from *Lactobacillus rhamnosus* (*LR*) KCTC 12202BP; this protein suppressed growth of CRC. We used DLD-1 cells to examine the mechanism by which p8 suppressed tumor growth and found that it exerted anti-proliferative and anti-migration activities. However, the anti-cancer properties were quite weak because p8 did not penetrate cells efficiently. Therefore, to improve delivery, we developed a gene therapy method to express the protein in cells directly [14].

The first attempt at gene therapy, along with the first demonstration of medical transfer of foreign genes into humans (not counting organ transplantation), was performed by Martin Cline in 1980 [15]. Gene therapy was approved by the National Institutes of Health in 1989 [16]. Since then, 2930 clinical trials have been conducted, with more than half of these (56.1%) in phase I by 2018 [17]. The first commercial gene therapy, Gendicine, was approved for clinical use against certain human cancers in 2003 [18]. Since the advent of genetic engineering technology, scientists have attempted to apply it in the context of human and veterinary medicine. Two main approaches are considered; the first of these is replacing or disrupting defective genes [19]. In the case of CRC, numerous genetic abnormalities and cumulative genetic changes trigger tumor development [20]. Thus, corrective gene therapy aims to repair the genetic abnormalities by introducing mutations. However, a major concern is the choice of target gene because the concept of correcting only a single gene may be flawed. Indeed, most tumors are likely caused by numerous genetic abnormalities [20,21,22]. The second approach is based on a viral-based delivery system for biopharmaceuticals such as proteins, peptides, and non-coding small RNAs. Most viruses can be engineered to express anti-cancer protein-coding genes; indeed, many recombinant viruses infect specific target cells and express anti-cancer proteins [23,24,25]. Logistically, these viral delivery systems are easier to develop and use than other immunotherapy strategies such as whole tumor cells [26]. In other words, viral delivery systems may be a more acceptable “off the shelf” vaccine vehicle given the relative ease of production, purification, and storage. An example of such a viral delivery system is adenovirus, which is a popular gene therapy vector due to its high transduction efficiency, broad cell tropism, high gene expression, and ease of production [27,28,29,30]. 

In this study, we examined the therapeutic potential of r-p8 protein and showed that it induced significant inhibition of tumor cell growth (by up to 20%) and migration (by up to 44%) when compared with a control; however, these values did not change even when the concentration was increased to 40 µM. Therefore, the efficacy of r-p8 in vitro suggested that it was not appropriate for use as a biopharmaceutical product, even though it showed low cellular toxicity. We wanted to find out why r-p8 exhibited such low anti-cancer activity against DLD-1 cells in vitro. Indeed, similar amounts of r-p8 were observed in DLD-1 cells, even when the concentration was increased to 10 µM. To overcome these limitations, we expressed p8 endogenously in a DLD-1 cell line. To do this, we developed a codon-optimized p8 gene for expression in mammalian cells. In vitro experiments revealed that the anti-cancer properties of endogenous p8 expression were 2-fold greater than those of exogenous r-p8 treatment (even at 40 µM). 

In summary, we showed that endogenous p8 expression was less toxic, more stable, and more efficient than exogenous r-p8 treatment; in addition, endogenous p8 expression showed strong cancer suppressive activity. Therefore, endogenous p8 expression may be a powerful form of gene therapy if it can be loaded onto cancer-specific viruses such as adenoviruses, adeno-associated viruses, or retroviruses.

## 2. Materials and Methods

### 2.1. Bacterial Strains and Culture

P8 protein was derived from *Lactobacillus rhamnosus* (*LR*) KCTC 12202BP, which was isolated from the human feces. *LR* is a probiotic and was obtained from the culture collection maintained at Cell Biotech Co., Ltd (Gimpo, Korea). The pCI-neo expression vector was used as a delivery vehicle for endogenous expression. Cells were cultured for 18–24 h at 37 °C in De Man, Rogosa and Sharpe agar (MRS) broth (Difco, Detroit, MI, USA). *Escherichia coli* (*E. coli*) strains DH5α and C41 (DE3) (Novagen, Madison, WI, USA) were cultured for 18–24 h at 37 °C in Luria-Bertani (LB) broth (Difco).

### 2.2. Construction of Codon-Optimized His-Tagged r-p8 Protein, and Expression and Purification in E. coli

The codon-optimized P8 gene harboring a hexa-histidine (6×His) tag and a Tobacco Etch Virus (TEV) protease cleavage site (305 bp) for *E. coli* cells was synthesized by Cosmogenetech, Inc. (Seoul, Korea) Table 1. The r-p8 protein was expressed from expression vector pET-28a. The p8 construct was transformed into *E. coli* strain C41 (DE3), which was cultured in M9 medium until the O.D. value reached 0.6. Overexpression of selenomethionine-substituted (SeMet) r-p8 was initiated by addition of 0.5 mM IPTG for 4 h. Cells were harvested and resuspended in 20 mM HEPES (pH 7.5)/150 mM NaCl. After sonication, the cell supernatant was obtained by centrifugation. The r-p8 protein was purified by binding to Ni^2+^-NTA agarose (Qiagen, Valencia, CA), followed by washing with 20 mM HEPES (pH 7.5)/150 mM NaCl/20 mM imidazole and elution with 20 mM HEPES (pH 7.5)/150 mM NaCl/300 mM imidazole. The 6×His tag was removed by TEV protease in the presence of 1 mM DTT. The homogeneity of the SeMet r-p8 protein was checked by size exclusion chromatography (HiLoad 26/60 Superdex 200 pg (GE Healthcare) equilibrated with 20 mM HEPES (pH 7.5)/150 mM NaCl).

### 2.3. Expression of Codon-Optimized r-p8 Protein in DLD-1 Cells

The *P8* gene codon that was optimized for expression in mammalian cells was synthesized by Cosmogenetech, Inc. The p8 DNA fragment (236 bp) was digested with *EcoR*I/*Not*I and cloned into the pCI-neo vector via the *EcoR*I/*Not*I site (Promega, Madison, WI) (Table 1). The construct was then transformed into *E. coli* DH5α for amplification. All restriction enzymes were purchased from New England BioLabs (Ipswich, MA). 

Colorectal cancer (DLD-1) cells were transfected with plasmid DNA (pCI-neo and pCI-neo-p8). The day before transfection, DLD-1 cells were plated in 6-well plates at a density of 7 × 10^5^ cells per well. After incubating overnight, cells were transfected using Lipofectamine 3000 (Invitrogen) in accordance with the manufacturer’s instructions [31]. The transfected cells were selected in RPMI 1640 containing antibiotics. (G-418) (Sigma, St. Louis, MO, USA).

### 2.4. Cell Culture

Human CRC cell line DLD-1 was purchased from the Korean Cell Line Bank (KCLB; Seoul, Korea) and maintained under 5% CO_2_/37 °C in Roswell Park Memorial Institute (RPMI)-1640 medium (Gibco, Grand Island, NY) containing 10% fetal bovine serum (Gibco) and 1% penicillin/streptomycin (Gibco). 

### 2.5. Cell Proliferation Assay 

DLD-1 cell lines (pCI-neo (EV) and pCI-neo-P8 (P8)) were seeded in 96-well plates (density, 1 × 10^3^ cells per well) and incubated at 37 °C. After 72 h, cell viability was determined in an MTT assay (Cell Counting Kit-8; Dojindo Laboratories, Tokyo, Japan). Absorbance was measured using a multifunctional microplate reader (SpectraMax M5; Molecular Devices, Sunnyvale, CA, USA). 

### 2.6. Wound Healing Assay 

DLD-1 cell lines (EV and P8) were seeded on 6-well plates (5 × 10^6^ cells per well). At 24 h post-seeding, the middle of the plate was scratched using a pipette tip. The cells were then washed three times with phosphate buffered saline (PBS) and incubated at 37 °C for 3 days. Wound healing was observed daily under a microscope (Nikon, Tokyo, Japan). 

### 2.7. ELISA Analysis 

The optimized ELISA procedure was performed as follows: 96-well polystyrene plates (SPL Life Sciences, Pocheon-si, Gyeonggi-do, Korea) were coated overnight at 4 °C with 100 μL diluted anti-p8 IgG (1:5500) (poly clonal-rabbit; Young In Frontier Co., Ltd, Seoul, Korea) in ELISA coating buffer (Bethyl Laboratories, Montgomery, TX, USA). Next, the wells were washed twice with 300 μL wash buffer (1× Tris-Buffered-Saline Buffer (TBS) with 0.05% Tween-20 (TBS-T)), followed by blocking with 300 μl blocking buffer (1× PBS and 5% Fetal Bovine Serum (FBS; Gibco)) for 1 h at room temperature (RT). The wells were washed three times with 300 μL wash buffer prior to addition of protein samples (nucleus extracts: 100 μL), followed by 150 min of incubation at RT. After sample binding, the wells were washed four times with 300 μL wash buffer (TBS-T), followed by addition of 100 μL anti-p8 IgG-biotin (Young In Frontier Co., Ltd) in 1 × PBS/5% FBS for 90 min at RT. Next, the wells were washed four times with 300 μL wash buffer (TBS-T), followed by addition of 100 μL streptavidin-HRP (166 pg/ml) (Young In Frontier Co., Ltd) in 1 × PBS/2.5% FBS for 30 min at RT. Next, the wells were washed four times with 300 μL wash buffer (TBS-T) followed by color development after addition of 100 μL tetramethylbenzidine (TMB) one solution (Bethyl Laboratories; Montgomery, TX, USA) for 20 min at RT in the dark. The reaction was stopped by addition of 50 μL stop buffer (Bethyl Laboratories). Absorbance was measured using a multifunctional microplate reader (SpectraMax M5; Molecular Devices). To construct a standard curve for r-p8, mouse sera (2-fold dilutions: 1000 ng mL^−1^ to 15.625 ng mL^−1^) was assayed in triplicate. Each sample was assayed at two different dilutions and run in duplicate. Results for endogenous p8 protein are reported as nanograms/milliliter.

### 2.8. Western Blot Analysis

DLD-1 cells were lysed in RIPA lysis buffer containing a protease inhibitor cocktail (Roche). Next, proteins (40 μg total) were separated by sodium dodecyl sulfate polyacrylamide gel electrophoresis (SDS-PAGE) and transferred to a polyvinylidene difluoride (PVDF) membrane (Amersham Bioscience, Piscataway, NJ, USA). Blotted membranes were blocked in 5% skimmed milk/T-TBS and then incubated overnight at 4°C with appropriate primary antibodies (Cell Signaling Technology, Danvers, MA, USA); all antibodies were diluted 1:1000. The membranes were washed three times (each for 15 min) with T-TBS and then blocked in 5% skimmed milk/T-TBS. The membranes were then incubated for 1 h at 4 °C with an HRP-linked secondary antibody (Cell Signaling Technology). Glyceraldehyde 3-phosphate dehydrogenase (GAPDH) was used as an internal control. Protein bands were detected using an enhanced chemiluminescence kit (Millipore, Billerica, MA, USA), followed by autoradiography using a Chemi-doc™ Touch Imaging System (Bio-Rad Laboratories, Hercules, CA, USA). 

### 2.9. Immunocytochemistry Using ImageXpress^®^ Micro Confocal Microscopy

Colorectal cancer (DLD-1) cells were seeded onto coverslips placed in 6-well plates. After 24 h, p8 protein (0–40 μM) was added to each well for a further 72 h. Cells were fixed for 15 min at RT in 3% paraformaldehyde (PFA) and then washed three times in PBS. Cells were permeabilized by incubation for 2 min in 0.2% Triton X-100/PBS and then washed. To reduce background signals, cells were blocked for 30 min with 4% bovine serum albumin (BSA) in PBS. Next, cells were incubated overnight at 4 °C with a rabbit polyclonal anti-p8 antibody (Young In Frontier Co., Ltd) or for 2 h at 4 °C with a mouse monoclonal anti-EpCAM antibody (Cell Signaling Technology). Protein localization was visualized using FITC-conjugated goat anti-rabbit IgG (Jackson ImmunoResearch Laboratories, Inc.; West Grove, PA, USA) and Alexa Fluor 568-conjugated donkey anti-mouse IgG (Invitrogen). For nuclear staining, cells were incubated for 1 h at RT with 5 µg mL^−1^ Hoechst 33,258 (Sigma), rinsed three times in PBS, and then mounted. Images were obtained under an ImageXpress® Micro Confocal microscope (Molecular Devices).

### 2.10. Flow Cytometry Analysis

To investigate the effects of endogenous p8 on the cell cycle phase distribution, the cells were analyzed by flow cytometry. DLD-1 cell lines (EV and P8) were plated and incubated for 48 h. The DLD-1 cells were removed from culture dishes by trypsinization, collected by centrifugation, and washed with PBS. 5 × 10^5^ cells from each sample were fixed in ice-cold 70% ethanol and incubated on ice for at least 30 min. Cells were then washed in PBS, resuspended in 400 μL PBS, and 50 μL RNAse (1 mg mL^−1^), and 50 μL propidium iodide (0.4 mg/ml) were added. After incubation (1 h at RT) the stained nuclei were analyzed with a flow cytometer (FACSCalibur, BD Biosciences, Glostrup, Denmark). Cell cycle distribution was analyzed.

## 3. Result

### 3.1. P8 Requires a Specific Delivery System

According to previous results [13], p8 shows anti-cancer properties that might act as a brake on the p53-p21-Cdk1/Cyclin B1 signaling pathway in DLD-1 cells, resulting in G_2_ arrest. However, exogenous r-p8 treatment exhibited low anti-cancer activity in vitro. 

**Figure 1:**
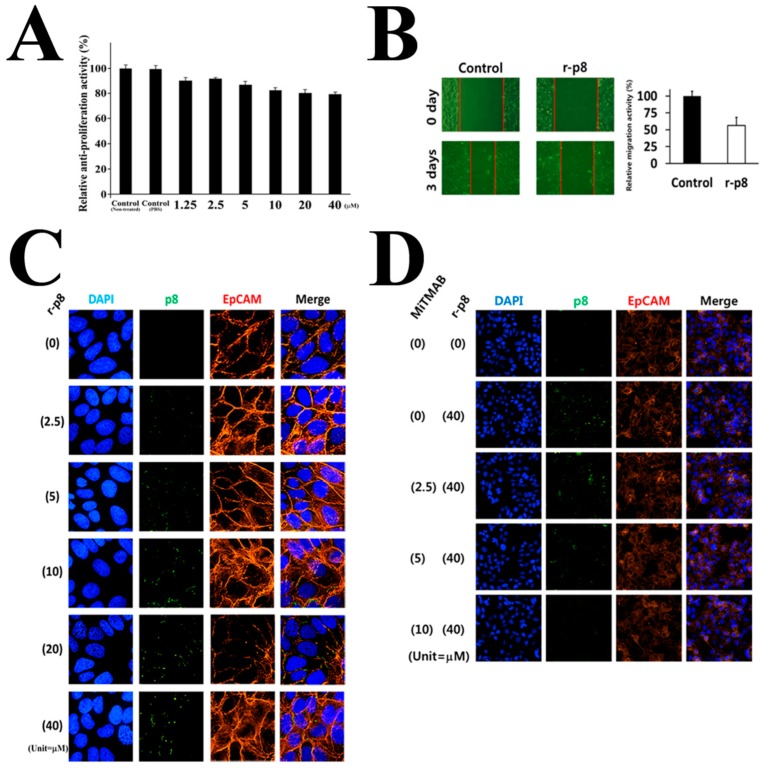
Characterization of p8 as an anti-cancer drug. Anti-cancer properties of exogenous r-p8 treatment. (**A**) R-p8 (0–40 μM) was incubated with DLD-1 cells (3 × 10^3^ cells/well) for 72 h, and anti-cancer efficacy was determined by MTT assay. (**B**) Anti-migration properties were examined in a wound healing assay. Wound healing was analyzed using Image J. (**C**) ImageXpress® Micro Confocal microscopy (60X) was used to determine the entry efficiency of r-p8. Entry of r-p8 into cells is concentration dependent. Cells were stained to detect r-p8 (Green), the cell membrane marker EpCAM (Red), or nuclei (DAPI: Blue). (**D**) ImageXpress® Micro Confocal microscopy (4X) was used to identify the route of entry used by r-p8. Cells were treated r-p8 (40 μM) with or without an endocytosis inhibitor (MiTMAB: 10 μM) and then stained to detect r-p8 (Green), the cell membrane marker EpCAM (Red), or nuclei (DAPI: Blue).

In this study, we examined the anti-cancer properties of exogenous r-p8 treatment in DLD-1 cells at various concentrations. Exogenous r-p8 treatment showed anti-proliferative (up to 20%) and anti-migration (up to 44%) activity (Figure 1A,B); these effects were only mildly dose-dependent (Figure 1A). 

**Figure 2:**
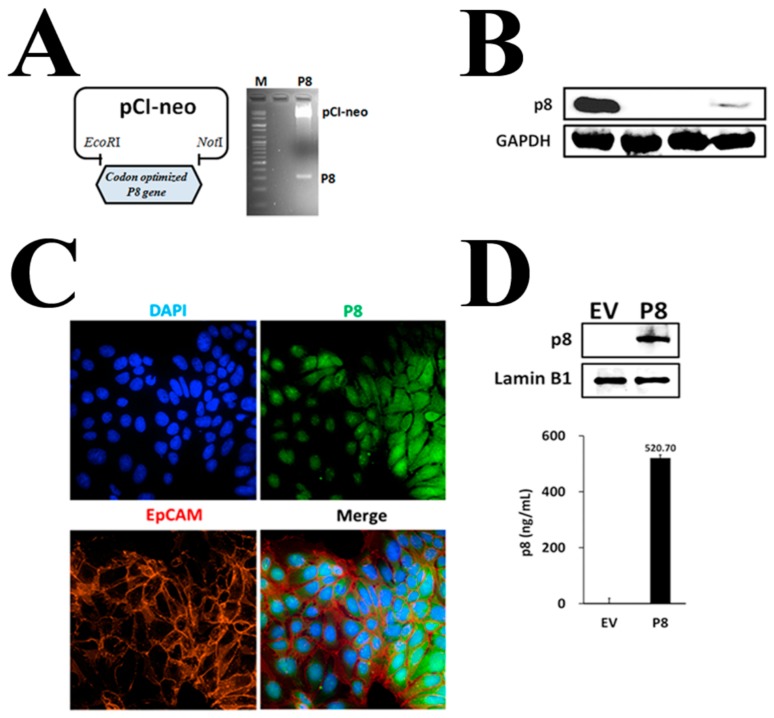
Endogenous p8 expression in DLD-1 cells. (**A**) The codon-optimized *P8* gene was cloned into the pCI-neo expression plasmid (*EcoR*I, *Not*I), and (**B**) endogenous expression of p8 was determined by western blotting [Lane 1: r-p8 (100 ng), lane 2: DLD-1 cell extract (30 μg), lane 3: EV cell line extract (30 μg), lane 4: P8 cell line extract (30 μg)]. GAPDH was used as an internal control. (**C**) Endogenous p8 expression was observed inside the cells by ImageXpress® Micro Confocal microscopy (60X). The cells were stained to detect p8 (Green), the cell membrane marker EpCAM (Red), or nuclei (DAPI: Blue). Endogenous expression of p8 in the nucleus was confirmed by (**D**) western blotting (upper panel) and ELISA (lower panel) of nuclear extracts. Lamin B1 was used as internal control.

Therefore, to find out why exogenous r-p8 treatment showed low anti-cancer activity and narrow dose-dependency, we used ImageXpress® Micro Confocal microscopy to examine its ability to penetrate cells. Little r-p8 protein was detected in the cytosol of DLD-1 cells, even at concentrations of 40 μM (Figure 1C). R-p8 is a probiotic-derived natural protein (8 kDa; height: 53 Å; width: 33 Å) and is not small enough to pass through mammalian cell membrane-pores or -ion channels. To evaluate endocytosis as a possible entry route, we observed translocation of r-p8 after exogenous treatment of cells with an endocytosis inhibitor (0–10 μM MiTMAB; Abcam, MA, USA) (Figure 1D). Inhibiting endocytosis led to a marked reduction in the amount of p8 within cells; this suggests that p8 enters cells via endocytosis. Uptake through endocytosis is a very inefficient route for drug delivery. Therefore, we asked whether increasing uptake would result in increased anti-cancer activity. To achieve this, we expressed r-p8 endogenously in mammalian cells using a pCI-neo vector (Figure 2A). We used codon-optimized sequences because some bacterial proteins fail to express properly in mammalian cells. We then measured expression of endogenous p8 by western blotting (Figure 2B) and visualized cellular localization using ImageXpress® Micro Confocal microscopy (Figure 2C). The endogenously expressed p8 proteins were observed in both the cytosol and nucleus (Figure 2C). Western blotting (above panel) and ELISA (below panel) of nuclear extracts confirmed that endogenously expressed p8 was translocated to the nucleus from the cytoplasm (Figure 2D, Figure S1).

### 3.2. Endogenous p8 Expression Showed Markedly Enhanced Anti-Cancer Activity

**Figure 3:**
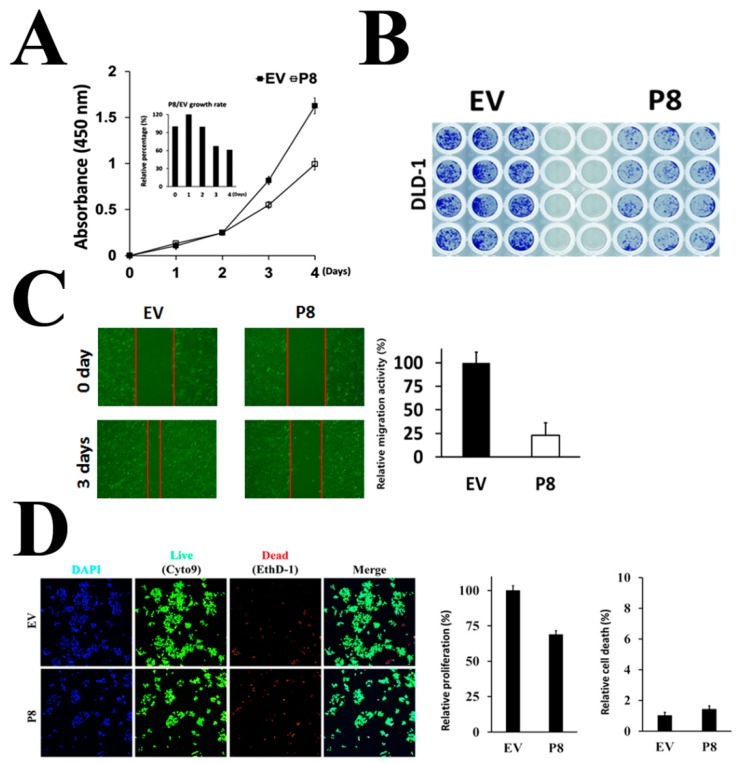
Endogenous p8 shows increased anti-cancer activity. (**A**) To examine whether endogenous expression improves the anti-cancer activity of endogenous p8 expression, we examined its anti-proliferative effects in an MTT assay. (**B**) Colony formation after endogenous expression of p8 was determined by staining with crystal violet. (**C**) The anti-migration activity of endogenous p8 expression was determined in a wound healing assay. Wound recovery was analyzed using Image J. (**D**) Anti-cancer efficacy of endogenous p8 expression was examined under an ImageXpress® Micro Confocal microscope. Cells were stained with the live/dead cell markers Syto9 (Green)/EthD-1 (Red) or with the total cell marker Hoechst (Blue).

To evaluate whether endogenous p8 expression increased its anti-cancer properties in vitro, we measured its ability to suppress proliferation of cancer cells (Figure 3). The results showed that endogenous p8 expression reduced tumor cell proliferation by ~40% (Figure 3A), which was up to 2-fold greater than that by 40 μM exogenous r-p8 treatment. Moreover, endogenous p8 expression suppressed colony formation (Figure 3B) and migration activities (Figure 3C) of DLD-1 cells compared with EV controls. Taken together, these results show that the anti-cancer activity of p8 is dependent on the amount that enters the cell. 

To determine the effects of p8 on various signal transduction pathways, we examined the signaling pathways associated with proliferation of susceptible phenotypes. First, we asked whether p8 induced apoptosis or cell cycle arrest in DLD-1 cells. The number of dead cells after treatment with 40 μM exogenous r-p8 was comparable with that after control (Figure S2); however, the number of dead cells did change after the concentration of exogenous r-p8 treatment was increased. Next, we examined the effect of endogenous p8 expression (Figure 3D). The number of dead cells in treated and control samples was similar, suggesting that endogenous p8 did not trigger apoptosis.

### 3.3. Effects of r-p8 on Anti-Cancer Signaling Pathways in DLD-1 Cells 

**Figure 4:**
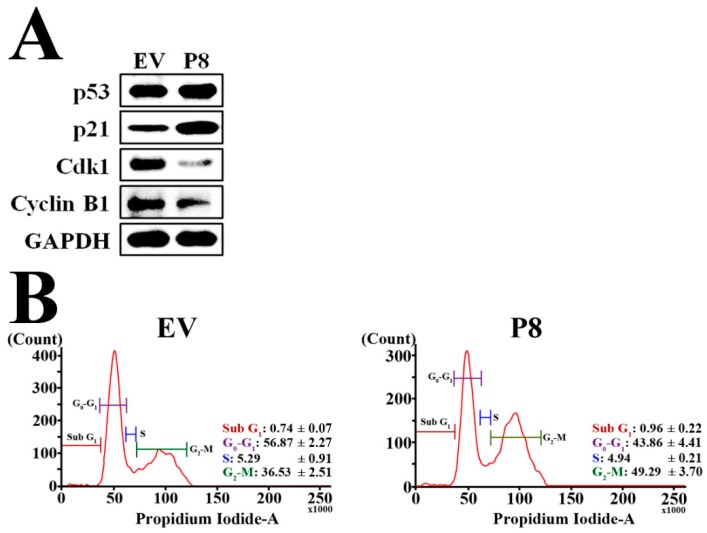
P8 targets anti-cancer signaling pathways in DLD-1 cells. (**A**) Western blot showing the effect of endogenous p8 expression on molecules associated with G_2_ arrest in DLD-1 cells (EV, empty vector). (**B**) To determine the effects of endogenous p8 expression on the cell cycle, cells were harvested and subjected to flow cytometry analysis. Endogenous r-p8 induced arrest of DLD-1 cells at G_2_ phase.

Finally, we investigated the effects of endogenous p8 expression on the cell cycle using western blotting to detect expression of cell cycle-related proteins (Figure 4A). We found that endogenous p8 expression strongly reduced expression of both Cyclin B1 and its partner protein Cdk1 in DLD-1 cells. We also found a marked increase in expression of p21, which suppresses Cyclin B1/Cdk1. Induction of p53 expression, a positive up-stream regulator of p21, was also observed. These data suggest that r-p8 might put a brake on the p53-p21 signaling pathway, resulting in arrest of DLD-1 cells at G_2_. To evaluate the effect of endogenous p8 expression on the cell cycle, we analyzed changes using flow cytometry (Figure 4B). Endogenous p8 expression induced marked growth arrest at G_2_. 

## 4. Discussion

Increased life expectancy means that cancers continue to be the leading cause of death among the elderly in many countries. Both the incidence and fatality rates of CRC have increased consistently in recent years [32,33]. Advanced-stage colon cancer is treated with chemotherapeutic regimens, which show poor response rates, severe side effects, and systemic toxicity [34,35]; this is because these potent drugs attack all cells that are replicating or dividing. 

Therefore, we need to develop novel anti-cancer drugs with minimal or no side effects. Biotherapeutic drugs such as antibodies and therapeutic proteins, including enzymes, are suitable candidates that have proven clinically effective in humans [36,37,38]. Previously, we reported a novel anti-cancer protein, p8, isolated from probiotic bacteria, which showed anti-cancer properties in vitro and in vivo; however, the anti-cancer activity was low [13].

Here, we provide evidence for these low anti-cancer properties. First, we found that the intracellular translocation of p8 is poor, with a narrow dose-dependent range of toxicity (Figure 1A–C). We hypothesized that the low penetration efficiency of p8 was due to the fact that as a probiotic-derived natural protein drug (8 kDa; height: 53 Å; width: 33 Å), it was too large to pass through pores or ion channels in the mammalian cell membrane (in which the pore size is less than ~25 Å). Therefore, we suspected that p8 may enter the cell cytosol by endocytosis; this was confirmed in in vitro assays (Figure 1C).

Therefore, we surmised that if the amount of p8 delivered to cells can be increased then it may show improved anti-cancer effects. To express p8 endogenously in mammalian cells, we generated a codon-optimized p8 gene and constructed a pCI-neo p8 expression plasmid (Figure 2A). We confirmed endogenous expression of r-p8 by western blotting and ImageXpress® Micro Confocal microscopy (Figure 2B,C). Indeed, r-p8 localized to both the cytosol and nucleus (Figure 2D); however, in the previous study, we failed to detect r-p8 in the nucleus in in vitro experiments [13]. These results suggest that r-p8 can penetrate the nucleus, even though we just failed to visualize it in the previous study. Importantly, the anti-cancer effects of endogenous p8 expression were 2-fold greater than those of exogenous r-p8 treatment at 40 µM (Figure 3).

Next, we asked how p8 suppresses growth of CRC cells. Figure 1B shows that neither exogenous nor endogenous p8 killed DLD-1 cells by inducing apoptosis. Therefore, we surmised that p8 suppresses CRC growth by inducing cell cycle arrest. We examined the expression level of factors related to the cell cycle and found that p8 inhibited the p53-p21-cyclin B1/Cdk1 pathway (Figure 4A). Furthermore, we used flow cytometry to confirm that p8 induced growth arrest at G_2_ (Figure 4B). 

In summary, we show that endogenous p8 expression suppresses growth of CRC cells by inhibiting Cdk1/Cyclin B1 activation via the p53-p21 pathway. Our ultimate goal is to develop a high efficiency delivery system for p8. Before using p8 as part of a viral delivery system, we needed to confirm several things. We now know that p8 can be expressed in mammalian cells and that endogenous p8 suppresses CRC growth more effectively than r-p8 treatment in vitro, and we have identified the mechanism underlying this suppression. Taken together, the results suggest that p8 is a strong candidate biopharmaceutical; the next step is to develop a CRC-specific viral vector harboring the p8 gene.

## Figures and Tables

**Table 1:** List of codon-optimized p8 gene segments and target organisms.

Target Organism/Mapping	Codon-Optimized Sequences	Size (bp)
Original *P8*	atggcaacagtagatcctgaaaagacattgtttctcgatgaaccaatgaacaaggtatttgactggagcaacagcgaagcacctgtacgtgatgcgctgtgggattattacatggaaaagaacagccgtgataccatcaagactgaagaagaaatgaaaccagtcctagacatgtccgacgatgaggtcaaagccctagcagaaaaggttctcaagaagtaa	222
*E. coli* cells/6×His-TEV-*P8* (*Nde*I/*EcoR*I)	catatgagaggatcgcatcaccatcaccatcac-attacgatatcccaacgaccgaaaacctgtattttcagggatcc-atggcaacagtagatcctgaaaagacattgtttctcgatgaaccaatgaacaaggtatttgactggagcaacagcgaagcacctgtccgtgatgcgctgtgggattattacatggaaaagaacagccgtgatactatcaagactgaagaagaaatgaaaccagtcctagacatgtccgacgacgaggtcaaagccctagcagaaaaggttctcaagaagtaggaattc	305
DLD-1 cells/*P8* (*EcoR*I/*Not*I)	gaattcatggctactgtcgacccagaaaaaaccctgttcttggacgaaccaatgaataaagtctttgattggtccaactctgaggccccggtacgggatgcgttgtgggattactacatggaaaaaaattccagggataccattaaaacagaagaagaaatgaagccagttctggacatgagtgacgacgaagtgaaagccctcgcggaaaaagttctcaagaaataggcggccgc	236

* Restriction enzyme sites are underlined.

## Supplementary Materials

The following are available online at https://www.mdpi.com/2073-4425/10/8/624/s1, Figure S1: Verification of no cytoplasmic contamination in the isolated nuclear fraction, Figure S2: Apoptotic property of exogenous r-p8 treatment.
