# Supplementary material for: Anti-Colorectal Cancer Effects of Probiotic-Derived p8 Protein
Source: Genes (Basel). 2019 Aug 19;10(8):624. doi: 10.3390/genes10080624 (PMC6723380; doi:10.3390/genes10080624)
Supplement: Supplementary file 1 [file genes-10-00624-s001.zip › genes-552049-SI.pdf]

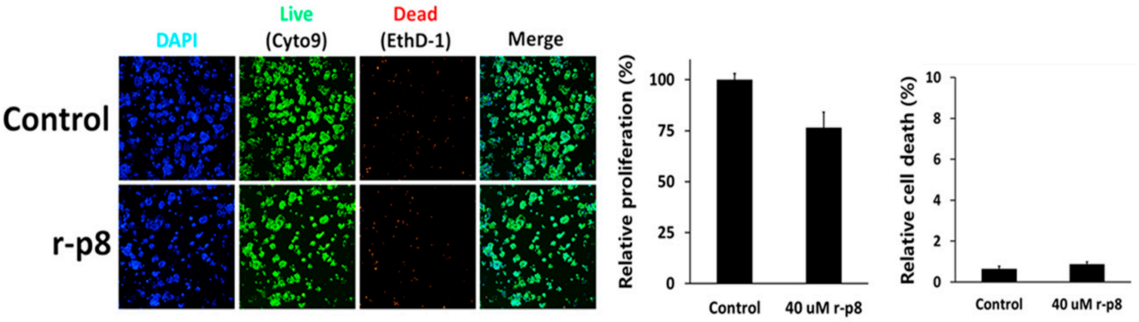

Figure S1. Verification of no cytoplasmic contamination in the isolated nuclear fraction. The cytoplasmic contamination in the isolated nuclear faction was determined by western blotting [Lane 1: DLD-1 cell lysate (30 μg), lane 2: cytoplasm fraction (30 μg), lane 3: nuclear fraction (30 μg)]. GAPDH was used as a cytoplasmic probe.

Figure S2. Apoptotic property of exogenous r-p8 treatment. R-p8 (40 μM) was incubated with DLD-1 cells (3 x 103 cells/well) for 72 h, and both cells [control and r-p8 (40 μM) treatment] were then stained with the Live/Dead cell markers Syto9 (Green)/EthD-1 (Red) or with the total cell marker Hoechst (Blue).
